# CD4^+ ^T cells spontaneously producing human immunodeficiency virus type I in breast milk from women with or without antiretroviral drugs

**DOI:** 10.1186/1742-4690-8-34

**Published:** 2011-05-13

**Authors:** Diane Valea, Edouard Tuaillon, Yassine Al Tabaa, François Rouet, Pierre-Alain Rubbo, Nicolas Meda, Vincent Foulongne, Karine Bollore, Nicolas Nagot, Philippe Van de Perre, Jean-Pierre Vendrell

**Affiliations:** 1 Laboratoire Virologie, Centre Muraz, Bobo-Dioulasso, Burkina-Faso; 2 Institut de Recherche en Science de la Santé/DRO, Bobo-Dioulasso, Burkina Faso; 3 Faculté de Pharmacie, 15 Avenue Charles Flahault, Montpellier 34060, France; 4 Laboratoire des Cellules Circulantes Rares Humaines (LCCRH) Institut de Recherche en Biothérapie, 80 Avenue Augustin Fliche, Montpellier 34295, France; 5 Laboratoire de Bactériologie-Virologie, CHU Lapeyronie,191 Avenue Doyen Giraud, Montpellier, 34295, France; 6 Département de Bactériologie-Virologie, Hôpital Lapeyronie, 191 Avenue Doyen Giraud, 34295 Montpellier cedex 5, France

## Abstract

**Background:**

Transmission of human immunodeficiency virus type 1 (HIV-1) through breast-feeding may involve both cell-free and cell-associated virus. This latter viral reservoir remains, however, to be fully explored. CD4^+ ^T cell-associated virus production in breast milk was therefore investigated.

**Methods:**

The *ex vivo *spontaneous production of HIV-1 antigen and HIV-1 RNA by CD4^+ ^T cells was measured in paired blood and breast milk samples from 15 HIV-1 infected women treated or not with antiretroviral drugs. Spontaneous antigen secreting cells (HIV-1-AgSCs) from breast milk and blood were enumerated by an ELISpot assay, and cell-associated HIV-1 RNA was quantified by real-time PCR in supernatants of CD4^+ ^T cells cultured for 18 hours without addition of polyclonal activators.

**Results:**

Among the CD4^+ ^T cells present in breast milk, memory cells expressing high levels of cell-surface activation markers were predominant. Spontaneous HIV-1-AgSCs were detected and enumerated in the breast milk of all 15 women, with a median number of 13.0 and 9.5 HIV-1- AgSCs/106 CD4^+ ^T cells in aviremic (n = 7) and viremic (n = 8) women, respectively. Cell- associated HIV-1 RNA was detected in cell-free supernatants from 4/7 aviremic and 5/8 viremic individuals at median levels of 190 and 245 copies/ml, respectively.

**Conclusions:**

Activated CD4^+ ^T cells producing HIV-1 are detected in the breast milk of untreated individuals as well as those receiving highly active antiretroviral therapy. This finding strongly suggests that HIV-1 replication occurs in latently infected CD4^+ ^T cells that, upon spontaneous activation, revert to productively infected cells. These cells might be responsible for a residual breast milk transmission despite maternal highly active antiretroviral therapy.

## Background

Today, while improvements have been made in prophylactic measures to prevent the perinatal transmission of HIV-1, its transmission through breastfeeding is still the cause of over half the estimated yearly 420,000 new pediatric infections worldwide [1]. Indeed, while it is universally recognized as the optimal source of nutrition and defense against disease in infants, breast milk is also a major mode of HIV-1 transmission from mother to child [2-4]. The mechanisms by which this occurs, however, remain poorly understood [5]. In breast milk, HIV-1 may be present in three different forms of potentially unequal transmission risk: (i) free virions measured as HIV-1 RNA, (ii) integrated provirus measured as HIV-1 DNA, and (iii) HIV-1 RNA that is released by activated cells that sustain the virus replication cycle and is measured as cell-associated HIV-1 RNA. High levels of free HIV-1 RNA in maternal plasma and in breast milk are associated with a high risk of breastfeeding transmission [6-11]. A similar association has been demonstrated with HIV-1 proviral DNA levels in breast milk, thus suggesting that both cell-free and cell-associated HIV-1s are involved in breastfeeding transmission [9,12-14]. Results of a study performed in Botswana suggest that up to 9 months postpartum, HIV-1 is mainly transmitted by cells containing the provirus while the cell-free virus is more frequently involved later on [15]. Furthermore, preliminary observations suggest that some babies breastfed by HIV-1 infected women taking antiretroviral therapy (ART) get infected despite undetectable levels of HIV-1 RNA in their mother's plasma or breast milk [16,17]. Importantly, the *in vitro *infectivity of the cell-associated virus has been found to be 100 to 1000 times higher than that of cell-free virus stocks [18]. Taken together, these observations strongly suggest that cell-associated virus is frequently involved in the transmission of HIV-1 by breastfeeding. HIV-1 persists in a latent form in resting CD4^+ ^T cells, even in patients receiving antiretroviral treatment (ART) and in whom the viral load is undetectable. These latently infected cells constitute a viral reservoir, which may be regarded as a cell type or anatomical site in which a functional form of the virus persists with increased stability compared to the pool of actively replicating virus [19]. A recent study shows that cell-free and, to a much lesser extent, cell-associated HIV-1 RNA levels in breast milk are suppressed by antiretroviral regimens used to prevent mother to child transmission, whereas no significant reduction in latently HIV-1 infected resting CD4^+ ^T cells is observed [20].

We recently demonstrated that breast milk contains such resting CD4^+ ^T lymphocytes and that these cells are capable of producing viral antigens (Ags) and virions after *in vitro *polyclonal-cell activation. In addition, these CD4^+ ^T lymphocytes showed a greater capacity to produce viral particles than their circulating blood counterparts [21]. Moreover, it has also been demonstrated that CD4^+ ^T cells from most viremic HIV-1 infected patients, spontaneously secrete HIV-1 virions as a consequence of an ongoing viral replication in the absence of ART or a residual HIV-1 replication under ART [22,23]. Thus, we hypothesized that breast milk contains CD4^+ ^T cells able to spontaneously produce HIV-1 proteins, RNA. and infectious particles.

In this study, we (i) characterized activated CD4^+ ^T cells in breast milk, (ii) enumerated CD4^+ ^T cells spontaneously producing HIV-1 antigens (HIV-1-AgSCs), and (iii) measured cell-associated HIV-1 RNA in cell-free supernatants in infected women treated or not with antiretroviral drugs. The human milk-derived activated CD4^+ ^T cells that spontaneously produced HIV-1 were barely affected by maternal antiretroviral therapy and might therefore be responsible for residual HIV-1 transmission.

## Results

### Study subjects

Women's characteristics, antiretroviral treatments and breast milk sample collection conditions are described in Table 1. According to national policy guidelines, 9 women received perinatal prophylactic treatment to prevent mother to child transmission of HIV-1, consisting of zidovudine given from between the 34th and 36th weeks of pregnancy until delivery plus a single dose of nevirapine during labor/delivery. The remaining 6 women were eligible for ART during pregnancy and received zidovudine, lamivudine and ritonavir-boosted lopinavir. The mean duration of ART until delivery was 36.4 days. Among the 15 women, the mean CD4^+ ^T cell count was 519 cells/mm3 and the mean plasma HIV-1 RNA level 13,105 copies/ml. Seven women, 5 treated with ART (nos. 1, 3, 9, 12 and 13) and two with the short perinatal prophylactic treatment (nos. 6 and 11), had undetectable plasma HIV-1 RNA load. The remaining seven women who received the short perinatal prophylactic treatment (nos. 2, 4, 5, 7, 10, 14 and 15) had a detectable plasma HIV-1 RNA load, and the one remaining woman receiving ART (no. 8) showed detectable viraemia. HIV-1 RNA was detected in the breast milk of five (35%) women; (mean 140 HIV-1 RNA copies/ml, range < 145-4,062 HIV-1 RNA copies/ml), four of whom had stopped ART at time of sampling and showed detectable HIV-1 plasma viral load.

**Table 1:** Characteristics of HIV-1 infected women

Patients no.	Initiation of antiretroviral treatment (days before delivery)	Duration of lactation until sampling (days)	Antiretroviral regimen	Treatment at time of sampling	**CD4^+ ^T cell counts/mm**^**3**^	HIV-1 RNA level (copies/ml)	
						
						plasma	Breast milk
1	15	54	ART^a^	Ongoing	NT	ND ^b^	NT
2	18	65	Short-course prophylaxis^c^	Withdrawal since 65 days	400	1776	ND
3	34	33	ART	Ongoing	762	ND	ND
4	35	11	Short-course prophylaxis	Withdrawal since 11 days	521	12,878	ND
5	38	14	Short-course prophylaxis	Withdrawal since 14 days	270	83,547	ND
6	26	55	Short-course prophylaxis	Withdrawal since 55 days	646	ND	ND
7	47	57	Short-course prophylaxis	Withdrawal since 57 days	658	6,790	ND
8	32	50	ART	Ongoing	305	34,937	4,062
9	17	29	ART	Ongoing	416	ND	ND
10	65	91	Short-course prophylaxis	Withdrawal since 91 days	628	50,036	772
11	58	77	Short-course prophylaxis	Withdrawal since 77 days	618	ND	190
12	15	52	ART	Ongoing	444	ND	ND
13	69	21	ART	Ongoing	533	ND	ND
14	46	9	Short-course prophylaxis	Withdrawal since 9 days	688	1,049	145
15	31	15	Short-course prophylaxis	Withdrawal since 15 days	384	4,526	308

^ a^ART, antiretroviral therapy.

^ b^Threshold: 300 copies/ml plasma and 60 copies/ml for breast milk.

^ c^Short-course perinatal prophylaxis (zidovudine until delivery and a single-dose of nevirapine during labor).

NT: not tested.

ND: not detected, < threshold.

### Characterization of CD4^+ ^T cells in breast milk

**Figure 1:**
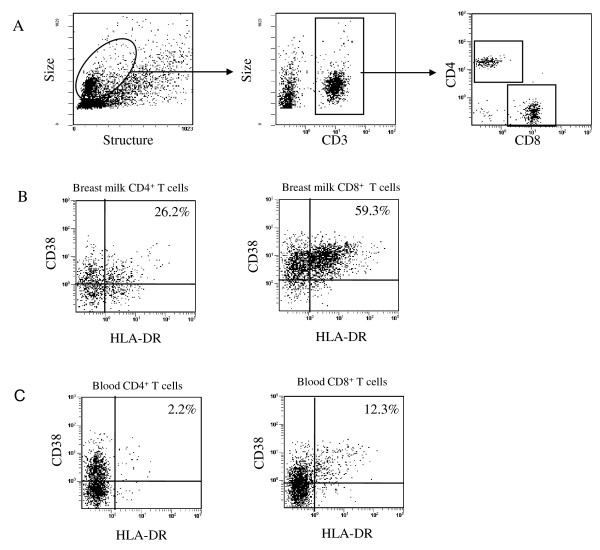
**Representative dot plots from breast milk and blood samples of an HIV-1-infected woman (no 8) (A) Gating strategy to explore breast milk CD4^+ ^T cells and CD8^+ ^T cells**. (**B**) Analysis of CD38 and HLA-DR cell-surface expression on breast milk CD4^+ ^T cells (left) and CD8^+ ^T cells (right). (**C**) CD38 and HLA-DR cell surface expression on blood CD4^+ ^T cells (left) and CD8^+ ^T cells (right) using the same gating strategy. The percentage of cells positive for both HLA-DR and CD38 staining is given in the upper quadrant of each dot plot.

As shown in one representative case (patient no. 10), we characterized the CD3^+^, CD4^+ ^and CD8^+^T cells as well as CD4^+ ^and CD8^+ ^T cells expressing HLA-DR and CD38 receptors in breast milk and blood by flow cytometry prior to CD4^+ ^T cell enrichment (Figure 1A, B, C). The CD4^+ ^T cells in the breast milk of 15 women represented on average 22.2% of the total T cell count, and the CD3^+ ^CD8^+ ^T cells represented 60.1%. A similar distribution was found in blood samples. The majority of CD4^+ ^and CD8^+ ^T cells in milk did not express the CD45RA receptors characteristic of naive T cells (mean 92.4% and 79%, respectively). The percentage of CD4^+ ^and CD8^+ ^T cells not expressing CD45RA was significantly lower in blood (mean 64.3% and 45.3%, respectively, *P *< 0.001). These results imply that the majority of T cells found in the milk are mainly memory T cells. This observation was confirmed by the high level of cell-surface CD45RO receptor expression on these cells (data not shown). In addition, as shown in Table 2, breast milk CD4^+ ^and CD8^+ ^T cells expressed higher levels of activation markers when compared with blood CD4^+ ^and CD8^+ ^T cells. Thus, breast milk from HIV-1 infected women contains predominantly activated memory CD4^+ ^T cells.

**Table 2:** Cell-surface marker expression on breast milk and blood T lymphocytes

Cell-surface marker	Breast milk	Blood	*P*
CD3^+ ^CD4^+^	22.2 (4.1-62.3) ^a^	29.2 (10.6-46.0)	NS^b^
CD3^+^CD8^+^	60.1 (18.7-83.4)	56.3 (39.1-82.7)	NS
CD4^+ ^CD45RA^-^	92.4 (64.2-98.1)	64.3 (43.4-88.1)	< 0.001
CD8^+ ^CD45RA^-^	79.0 (69.6-99.3)	45.4 (25.3-72.5)	0.003
CD4^+ ^HLA-DR^+^	42.6 (19.2-87.5)	12.0 (1.0-18.1)	0.004
CD4^+ ^CD38^+^	39.2 (22.1-72.8)	51.3 (24.5-81.2)	NS
CD4^+ ^CD38^+ ^HLA-DR^+^	23.3 (12.6-46.6)	8.1 (0.3-15.3)	0.01
CD8^+ ^HLA-DR^+^	76.4 (24.5-89.2)	20.6 (11.5-45.9)	< 0.001
CD8^+ ^CD38^+^	92.5 (45.4-98.2)	54.2 (27.2-99.8)	< 0.001
CD8^+ ^CD38^+ ^HLA-DR^+^	72.3 (16.3-95.6)	11.7 (9.3-43.2)	< 0.001

^a^mean (range).

^b^NS, not significant

### Enumeration of HIV-1-AgSCs in breast milk and blood derived CD4^+ ^T cells

**Figure 2:**
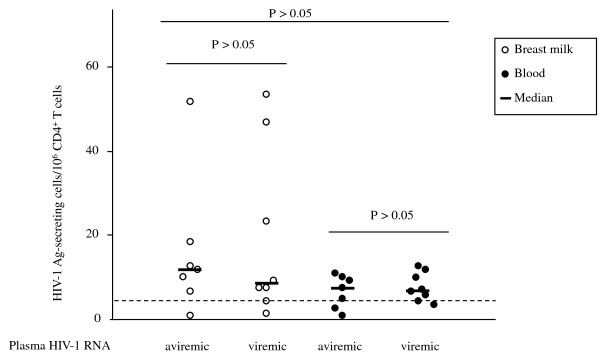
**Detection of *ex vivo *HIV-1 Ag secreting CD4^+ ^T lymphocytes in breast milk and blood**. HIV-1 infected CD4^+ ^T cells able to spontaneously produce HIV-1 Ag were enumerated by an ELISpot assay aimed at detecting p24 secretion. Spontaneous HIV-1-AgSCs were detected in breast milk cell samples from all the women tested. Dotted line indicates the lower limit of quantification of the test (3 HIV-1-AgSCs/10^6 ^CD4^+ ^T cells). The number of HIV-1-AgSCs showed no significant difference between individuals in whom plasma HIV-1 RNA was detectable or not nor was any difference found between breast milk and blood compartments (Mann Whitney U test, P > 0.05).

To evaluate the ability of the CD4^+ ^T lymphocytes to spontaneously secrete HIV-1 Ag and viral particles, freshly purified CD4^+ ^T cells from paired breast milk and blood samples were directly tested using our ELISpot assay. HIV-1-AgSCs were detected in breast milk cells from all women. As shown in Figure 2, the median number of HIV-1-AgSCs was similar in aviremic (*n *= 7) and viremic (*n *= 8) subjects, 13.0 HIV-1-AgSCs/10^6 ^CD4^+ ^T cells [Interquartile Range (IQR) 9.5-16.5 HIV-1- AgSCs/10^6 ^CD4^+ ^T cells] and 9.5 HIV-1-AgSCs/10^6 ^CD4^+ ^T cells (IQR 8.1-29.4 HIV-1-AgSCs/10^6 ^CD4^+ ^T cells), respectively (P > 0.05). HIV-1-AgSCs were also detected in the blood of viremic and aviremic women, median, 8.1/10^6 ^CD4^+ ^T cells (IQR, 4.0-9.5/10^6 ^CD4^+ ^T cells) and 6.25/10^6 ^CD4^+ ^T cells (IQR, 5.4-10.3/10^6 ^CD4^+ ^T cells, respectively), the numbers of which showed no significant difference between the two groups (*P *> 0.05).

### Detection of cell-associated HIV-1 RNA in supernatants from breast milk- and blood-derived CD4^+ ^T cell cultures

**Figure 3:**
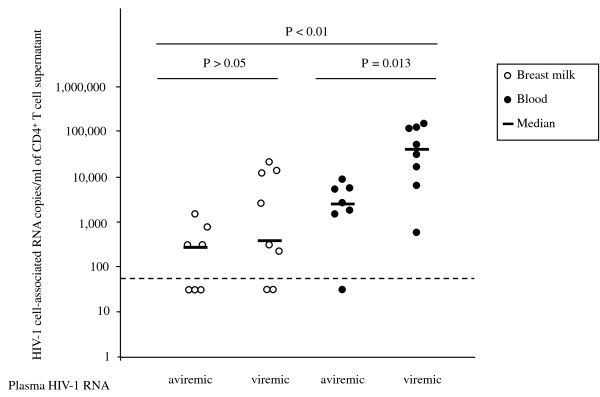
**Cell-associated HIV-1 RNA from breast milk and blood derived CD4^+ ^T cell culture supernatants**. HIV-1 RNA was quantitated in cell-free culture supernatant following 18 hours of incubation. Results from breast milk and blood cells were separated according to the detection of plasma HIV-1 RNA. Dotted line indicates the lower limit of quantification of the test (60 HIV-1 RNA copies/ml). The cell-associated HIV-1 RNA levels were similar between aviremic and viremic individuals in breast milk-derived cells but were lower in blood-derived cells from aviremic individuals by comparison with viremic individuals (Mann Whitney U test).

HIV-1 RNA was also quantified in the culture supernatant following 18 hours culturing of breast milk- and blood-derived CD4^+ ^T cells. As shown in Figure 3, concerning the breast milk samples, breast milk cell-associated HIV-1 RNA was detectable in 10 of the 15 subjects (66.7%), the HIV-1 RNA levels were similar in women with detectable or undetectable plasma viral load: median, 245 RNA copies/ml (IQR, 113-12,300 RNA copies/ml) and 190 RNA copies/ml (IQR, 30-261 copies/ml), respectively. No correlation was observed between the number of HIV-1 RNA copies detected in the supernatants and the number of HIV-1-AgSCs. These data suggest that the presence of cells spontaneously producing HIV-1 RNA in breast milk is independent of plasma HIV-1 RNA levels. In blood samples, cell-associated HIV-1 RNA was detected in 14/15 individuals (93.3%) with a median level of 2,261 RNA copies/ml (IQR, 1,629-5,190 RNA copies/ml) in aviremic women (range 583-119,981) and 13,855 (IQR, 40,051-111,390 RNA copies/ml) in viremic women. Unexpectedly, although a similar number of HIV-1-AgSCs was found in the breast milk of aviremic and viremic women, the cell-associated HIV-1 RNA copies were significantly higher in the women with detectable viral load (*P *< 0.01). CD4^+ ^T cell-associated HIV-1 RNA levels were significantly higher in blood than in breast milk (*P *< 0.01). In subjects with undetectable HIV-1 viral load in plasma and breast milk (n = 5), both cell-associated HIV-1 RNA and HIV-1-AgSCs were detected in the breast milk, suggesting that the antiretroviral treatment was not fully effective at suppressing spontaneous virus production in breast milk.

### In vitro infection of CD4^+ ^T cells using breast milk- and blood-cell culture supernatants

**Figure 4:**
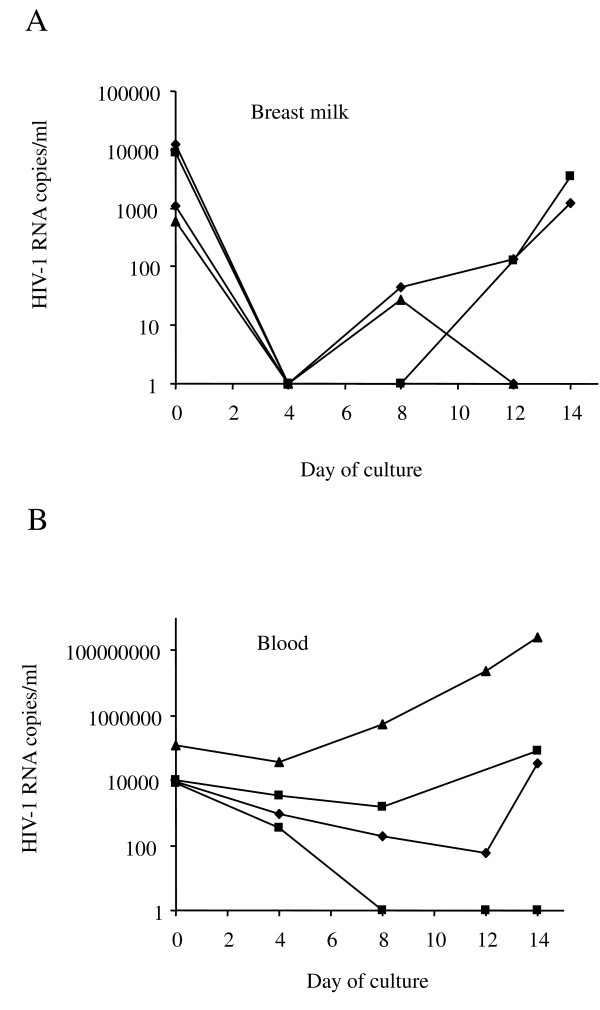
**Co-culture of breast milk- and blood-cell viral-culture supernatants with CD4^+ ^T cells**. The infectivity of virus secreted into culture supernatants was tested after 18 h of incubation by co culturing with phytohemagglutinin-activated CD4^+ ^T cells from healthy blood donors. **A**) HIV-1 RNA quantification in CD4^+ ^T co-culture with breast milk cell supernatants. **B**) HIV-1 RNA quantification in CD4^+ ^T co-culture with blood cell supernatants.

The infectivity of the virus secreted in breast milk- and blood- cell culture supernatants was assessed by infection of *in vitro *activated CD4^+ ^T cells provided by healthy blood donors. As shown in Figure 4, a decrease in HIV-1 RNA levels, followed by a sustained rebound of HIV-1 RNA, was observed in three blood-derived supernatants and two breast milk-derived supernatants, demonstrating the infectiousness of the virus. Successful *in vitro *infections were obtained using samples from women not receiving ART. The resulting supernatant fluids exhibited a viral load of over 10,000 copies/ml after 18 hours of CD4^+ ^T cell incubation. Within the first few days of *in vitro *infection, we observed a decrease in HIV-1 viral load in the breast milk derived supernatant. This may be related to the membrane fixation and entry of the HIV-1 into the target cells before completion of the virus cycle. The decline in viral load appears less visible during the first few days of target cell culture with blood-derived compared to breast milk-derived supernatant. This may be related to the higher HIV-1 viral load in blood supernatant for the same number of target CD4^+ ^cells.

### Quantification of HIV-1 DNA in breast milk- and blood-derived CD4^+ ^T cells

HIV-1-proviral DNA was measured in 12 of the 15 breast milk samples. The median HIV-1 DNA level was 3,178 DNA copies/10^6 ^CD4^+ ^T cells (IQR, 460-23,646 DNA copies/10^6 ^CD4^+ ^T cells) and showed no significant difference between aviremic- and viremic-women. HIV-1 DNA was also detected in the circulating CD4^+ ^T cells of the same 12 subjects, median 23,310 copies/10^6 ^CD4^+ ^T cells (IQR, 1,875-117,886 copies/10^6 ^CD4^+ ^T cells), again with no significant difference between aviremic versus viremic subjects.

## Discussion

To investigate the cells potentially involved in HIV-1 postnatal transmission through breastfeeding, freshly purified breast milk CD4^+ ^T cells were enumerated and characterized for their capacity to spontaneously produce HIV-1 Ag, using a sensitive HIV-1 Ag ELISpot assay. In parallel, after an overnight cell-culture step, cell-associated HIV-1 RNA levels were measured in cell culture supernatants.

We have demonstrated that the majority of breast milk CD4^+ ^T cells express low levels of CD45RA receptors and, concomitantly, high levels of HLA-DR and CD38 markers thus allowing them to be considered as being activated. While liquid nitrogen conservation and thawing may slightly modify the expression of cell surface markers on T lymphocytes [24], this cannot account for the large differences observed between CD4^+ ^T cells derived from blood and those from breast milk. In addition, the level of CD38 and HLA-DR expression observed in this study were 194 similar to those previously observed in fresh blood- and breast milk-derived T cells [25]. These data clearly indicate that a large fraction of CD4^+ ^T cells present in the breast milk of HIV-1 infected women comprise activated memory T cells. This is consistent with the physiological role of breast milk as a source of immunologically active cells [21,25-27], and suggests a minimal, if any, blood CD4^+ ^T cell contamination since the peripheral CD4^+ ^T cells are mainly naive. Breast milk lymphocytes may become highly activated during extravasation and/or transepithelial migration, or by exposure to the cytokines and chemokines contained in the breast milk micro environment [28-31].

In the second step of our study, freshly purified CD4^+ ^T cells from paired breast milk and blood samples of HIV-1 infected women were assayed for cell associated virus production using an ELISpot assay to analyze p24 secretions at the single cell level. We also quantified HIV-1 RNA levels after a short period of culture of CD4^+ ^T cells without the addition of polyclonal activators. We and others have previously shown that HIV-1 latently infected CD4^+ ^T cells derived from blood and breast milk are able to sustain the viral cycle and produce viral antigens and virions, following their polyclonal activation *in vitro *[21,32-34]. *In vivo*, some of the HIV-1 latently infected breast milk-derived CD4^+ ^T cells may revert to productively infected lymphoblasts if they are able to survive for an extended period of time in the gut or body of the infant. However, in subjects untreated by ARV, the vast majority of the virus is produced by activated CD4^+ ^T cells that play a key role in HIV-1 transmission. These cells have a very short half-life, surviving only about 1 day before dying as the result of viral cytopathic effects or the host cytolytic effector response. The present study provides evidence of the existence of HIV-1 productively infected cells in breast milk. P24 and HIV-1 secretion were detectable after only a short period of culture demonstrating that these breast milk-derived activated CD4^+ ^T cells constitute a replication-competent form of the HIV-1 cell reservoir. Given the fact that a majority of CD4^+ ^T cells in breast milk are physiologically activated in HIV-1 infected individuals, we hypothesized that some of the breast milk-derived T cells latently infected by HIV-1 revert to productively infected cells upon activation in the mammary gland. This could explain why HIV-1-AgSCs were found in the breast milk of all the women tested. As the number of immunospots (each one representing one HIV-1-secreting CD4^+ ^T cell) was similar in aviremic and viremic women, we can infer that the presence of HIV-1-AgSCs in breast milk is not related to plasma HIV-1 load. We assume that our observations reflect the particular dynamics of HIV-1 replication within the mammary gland and the existence of a functional reservoir probably involved in virus transmission through breast-feeding. On the other hand, the culture conditions used in this study cannot be considered as representative of the complex network involved in breast milk transmission that includes the gut and MALT of the infants. Cytotoxic T lymphocytes are associated with the control of HIV-1 and SIV viremia [35]. Studies have demonstrated that HIV-1-specific cytotoxic CD8^+ ^T cells are present in the breast milk of infected women where they may have a critical role of limiting HIV-1 replication within the mammary gland [25]. The depletion of CD8^+ ^T cells performed in our study likely diminished any HIV-1 specific response and therefore potentially facilitated the HIV-1 secretion *in vitro*.

The fact that HIV-1-AgSCs were also detected in breast milk samples with undetectable HIV-1 RNA suggests that HIV-1-AgSCs release insufficient levels of HIV-1 RNA for detection and/or that the time of transit of these cells into the breast milk is too short to allow HIV-1 RNA to be detected in breast milk. In women with successful ART, undetectable HIV-1 RNA in both plasma and breast milk has been interpreted as reflecting the cessation of viral replication within maternal lymphoid tissues [36,37] along with that in the mammary gland [38]. All but one woman receiving ART had undetectable plasma and breast milk HIV-1 RNA loads indicating the effectiveness of treatments on cell free HIV-1. The high viral load observed in plasma from one woman (no. 8) after more than 2 months of ART suggests a default in observance or the development of HIV-1 resistance to antiretroviral drugs. While ART has been associated with a dramatic decrease in HIV-1 RNA levels and, to a lesser extent, in HIV-1 DNA levels [12] in blood and breast milk, its impact on cell associated HIV-1 RNA has been proposed as being only moderate [20]. This cell-associated HIV-1 RNA might therefore constitute a source of HIV-1 transmitted by breastfeeding women successfully treated with ART [16].

In the third experimental step, we showed that cells characterized and enumerated by the HIV-1- Ag ELISpot assay also secrete HIV-1 particles, since the majority of breast milk and blood culture supernatants contained infectious HIV-1 RNA. Surprisingly, the levels of HIV-1 RNA were significantly higher in supernatants of cultured blood CD4^+ ^T cells as compared to cultured breast milk CD4^+ ^T cells, particularly in HIV-1 viremic individuals. Pretreatment of CD4^+ ^T cells with pronase before testing to characterize the cellular HIV-1 RNA secretion [22] revealed that blood CD4^+ ^T cells passively release high levels of cell-bound membrane HIV-1 particles upon incubation. In addition, the *ex vivo *detection of cell-associated HIV-1 RNA in the blood of aviremic individuals suggests a residual virus replication despite undetectable HIV-1 plasma viral load. This observation is in agreement with data clearly indicating that CD4^+ ^T cells in which HIV-1 transcription occurs persist in peripheral blood mononuclear cells from patients receiving potent antiretroviral therapy [22,34,39,40].

We hypothesized that the HIV-1-AgSCs and cell-associated HIV-1 RNA detected in breast milk from women on ART reflected the production of virus from stable reservoirs, such as the latent reservoir of resting CD4^+ ^T cells and perhaps macrophages. It can also be reasonably assumed that HIV-1-AgSCs and cell-associated HIV-1 RNA do not originate from additional viral replication owing to the suppressive effect of ART. The virus produced by the HIV-1 infected latent CD4^+ ^T cells becomes detectable as cell-associated virus, but not as cell-free virus, because of the low lymphocyte content of breast milk. According to our results and considering the estimated daily breast milk consumption [41], an infant breastfed by an HIV-1-infected woman may ingest an average of 178 HIV-1-AgSCs per day during his/her first four months of life. As one HIV-1- replicating cell releases at least 1,000 viral particles [18,33], the infant daily exposure could be around 178,000 cell-associated HIV-1 RNA. Thus, babies fed on breast milk containing no detectable cell-free virus may have their mucosa exposed to high levels of HIV-1 particles spontaneously secreted by HIV-1 infected CD4^+ ^T cells. The HIV-1-AgSCs described here may access the infant's tissues given that previous studies have shown immunologically active cells from breast milk infiltrating the tissues of the intestinal tract of the recipient [26,42,43]. Cell associated viral particles in contact with mucosa may penetrate to the submucosa through musal breaches or via transcytosis through specific molecular scaffolds and the molecular machinery of epithelial cells [44].

Our data reinforce the findings of several previous studies suggesting that latently HIV-1 infected cells are an important source of mother to child-transmission [9,14,15,20,45]. The ability of short-course antiretroviral regimens to reduce the breast milk transmission could be explained by effects of treatment on infectious virions [46-51]. In contrast, ART may prove to be poorly efficient at controlling cell-associated viral transmission since: (i) cell-associated HIV-1 RNA levels in breast milk are only modestly affected by ART [20], and (ii) we detected HIV-1-AgSCs and cell-associated HIV-1 RNA in women with undetectable HIV-1 plasma viral load. *In vitro *infection of donor cells indicated that the virus particles secreted into the cell culture supernatants from breast milk cells are infectious. Taking into account the low bioavailability of ritonavir/lopinavir in breast milk [52], we assume that these protease inhibitors are unable to suppress the release of infective virus from the HIV-1 cell reservoir in women receiving protease inhibitor-containing regimens. By contrast, while most reverse transcriptase inhibitors have a good bioavailability in breast milk, they are only efficient on viruses undergoing new cycles of infection. Thus, reverse transcriptase inhibitors would not be effective at controlling viruses produced from a stable reservoir. Recent results demonstrated that HIV-1 transmission to breastfed babies is decreased but not eliminated by maternal ART [48-53]. These observations may reflect the interrelationship between HIV-1 cell reservoirs, T cell activation, and antiretroviral bioavailability in breast milk.

In conclusion, our study has shown that most CD4^+ ^T cells in the breast milk of HIV-1 infected women are activated memory cells, some of which are able to spontaneously produce HIV-1 antigens and virions in the absence of in *vitro *activation. In women successfully treated by ART during lactation, these cells can be detected in both blood and breast milk despite undetectable levels of HIV-1 RNA in these compartments. These results suggest that ART administered to HIV-1 infected women during lactation is ineffective at suppressing cell-associated virus replication and thus may incompletely inhibit the breastfeeding transmission of HIV-1. The evaluation of alternative prevention strategies against the breastfeeding transmission of HIV-1 from infected mothers, such as physical or chemical treatment of extracted maternal milk or infant antiretroviral prophylactic treatment throughout the breastfeeding period needs consideration.

## Methods

### Study population and sample collection

This study was conducted at the Centre Muraz, Bobo-Dioulasso, Burkina Faso and at the University of Montpellier 1, France. The study was approved by the Ethical Committee of the Centre Muraz and the National Ethical Committee of the Ministry of Health, Burkina Faso, and written informed consent was obtained from all participants. Fifteen HIV-1 infected lactating women volunteered to participate. The mean duration of lactation was 42.2 days (range 9-91 days). Immediately after a feed, each woman provided 70 ml of breast milk, by bimanual expression directly into a sterile polypropylene tube, as well as 20 ml of blood. Plasma HIV-1 RNA levels were measured in the Centre Muraz, Bobo-Dioulasso using the Generic HIV Charge Viral kit, (Biocentric, Bandol, France) and ABI PRISM^® ^7000 thermocycler (Applied Biosystems, Foster City, USA) [32]. The lower limit of quantification (LLQ) of the test was 300 HIV-1 RNA copies/ml. Fresh blood CD4^+ ^T lymphocytes were enumerated by flow cytometry (Becton Dickinson, BD Bioscience, and San Jose, CA).

### Isolation of breast milk CD4^+ ^T cells

Breast milk cells were separated as previously described [54]. Breast milk samples were processed within 4 h of collection. The acellular fraction (lactoserum and lipid fraction) was removed by centrifugation at 1,200 g for 15 min. Breast milk cell pellets were washed three times in PBS supplemented with 5% fetal calf serum (FCS) and finally suspended in RPMI 1640 medium plus 10% FCS, 2 mM L-glutamine, 100 U/ml penicillin, and 100 μg/ml streptomycin (complete medium, all reagents from Eurobio). At least one fifth of the collected breast milk cells were stored in liquid nitrogen before flow cytometry analysis and the remaining cells were used for CD4^+ ^T cell purification. Breast milk- and blood-derived CD4^+ ^T lymphocytes were isolated by negative selection using an immunorosetting method (Rosette SepTM CD4 cell enrichment cocktail, Stemcell Technologies). The cocktail used allows the cross linking of unwanted leukocytes with red blood cells using antibodies directed against CD8, CD16, CD19, CD36, CD56 and glycophorin A. Red blood cells were prepared from 5 ml of whole blood from healthy donors by centrifugation of the sample for 10 min at 50 × g. They were then washed three times in PBS-2% FCS before being re-suspended in 1 ml of PBS-2% FCS. Red blood cell concentrates were kept at 4°C for 15 days to discard residual blood leukocytes before being added to the breast milk cell suspension. Red blood cells were then added to 3 ml of the breast milk cell suspension. When centrifuged over the buoyant density medium, rosetted cells were pelleted along with red blood cells. The enriched CD4^+^T cells were recovered from the Ficoll-plasma interface, washed three times in PBS/2% FCS and re-suspended at a final concentration of 1 × 10^5^cells/ml in culture complete medium. This method resulted in the elimination of 95% of non-T CD4^+ ^lymphocytes [54].

### Isolation of blood CD4^+ ^T cells

Blood CD4^+ ^T cells were purified using an immunorosetting method (Rosette SepTM CD4^+ ^cell enrichment cocktail, Stemcell Technologies) [32]. Purified CD4^+ ^T cells were suspended in complete culture medium at a final concentration of 1 × 10^6 ^cells/ml.

### Flow cytometry analysis

The phenotypic characterization of breast milk and peripheral blood mononuclear cells stored in liquid nitrogen was performed in the Montpellier laboratory using Abs conjugated to fluorescein isothiocyanate (FITC), phycoerythrin (PE/RD1), energy coupled dye (ECD), or phycoerythrin cyanine 5 (PC5) directed to CD3, CD4, CD8, CD38, CD45RA and HLA-DR cell-membrane markers (Beckman-Coulter, Fullerton, CA). Stained cells were analyzed using a FC-500 flow cytometer (Beckman-Coulter). The breast milk and blood T cell analyses were based on a forward versus side scatter histogram and CD3 positive events. Our design was to run 1,000 gated T cells. Percentages of CD4^+ ^and CD8^+ ^T cells in breast milk and blood lymphocytes were estimated as the percentage of CD3 positive events in the CD3-PC5 size histogram gate. The spontaneously activated CD4^+ ^T cell subset from breast milk was defined as the CD3^+^, CD4^+^, CD45RA- T cells expressing HLA-DR and CD38 cell-surface markers.

### HIV-1-Ag ELISpot assay

Immobilon-P membrane 96-well plates (MAIPN 4550, Millipore Corporation, Bedford, MA) were coated overnight at 4°C with 100 μl of a mixture of anti-HIV-1 polyclonal Abs prepared as previously described [32]. To enumerate p24 spontaneously secreting cells, enriched CD4^+ ^T cells from breast milk and blood were seeded on the nitrocellulose plate for 18 h, at a concentration of 1 × 10^5 ^CD4^+ ^T cells/100 μl per well. Immunospots were analyzed and counted in the Montpellier laboratory by video camera imaging and computer-assisted analysis (KS ELISPOT, Carl Zeiss, Jena, Germany), each spot representing the fingerprint of one HIV-1-antigen secreting cell (HIV-1- AgSC). Results were expressed as the number of spots read/10^6 ^CD4^+ ^T cells tested. The threshold for the lower limit of detection of HIV-1 Ag cell secretion in this assay was established using mean values obtained by testing breast milk and blood samples from 10 healthy controls uninfected by HIV-1 (mean + 2SD = 3 immunospots/10^6 ^cells).

### Quantification of cell-free and cell-associated HIV-1 RNA levels

Cell-associated HIV-1 RNA secretion was explored by the quantification of HIV-1 RNA secreted by CD4^+ ^T cells after a short culture period. Cell free virus was investigated by measuring the HIV-1 RNA viral load in lactoserum and plasma. Cell-free and cell-associated HIV-1 RNA extraction from lactoserum/plasma and 18 h cell culture supernatants stored at -80°C were performed in the Montpellier laboratory using the High Pure Viral RNA Kit (Roche Diagnostics, Indianapolis, IN), according to the manufacturer's instructions. Samples were centrifuged 1 hour at 23,500 × *g *at 4°C before RNA extraction. With this ultrasensitive protocol, the LLQ was 60 HIV-1 RNA copies/ml. Supernatants from 5 HIV-1 uninfected controls were below this threshold.

### Co-culturing of viral-culture supernatants with donor CD4^+ ^T cells

The infectivity of virus secreted into culture supernatants was tested after 18 h of incubation by coculturing with CD4^+ ^T cells. Target CD4^+ ^T cells were obtained from healthy blood donors within 24 h of donation. CD4^+ ^T cells were enriched from whole blood by negative selection as described above and were activated for 48 hours with phytohemagglutinin (4 μg/ml) plus 10 U/mL recombinant human IL-2 (Invitrogen, Grand Island, NY) in complete culture medium. CD4^+ ^T cells from three different donors were combined for each culture. Then, 2 × 10^5 ^activated CD4^+ ^T cells in 100 μl of culture media were cultivated with 100 μl of supernatant for 14 days. Twice a week, culture medium above the settled CD4^+ ^T cell was removed for HIV-1 RNA detection and replaced with an equal volume of complete culture medium.

### Quantification of cell-associated HIV-1 DNA

After 18 hours of incubation, CD4^+ ^T cells were collected and stored in liquid nitrogen in the Montpellier laboratory before measuring the HIV-1 DNA levels using an in-house real time PCR assay as previously described [55]. To determine precisely the amount of DNA in the purified CD4^+ ^T cells, all samples were tested using LightCycler-Control kit DNA (Roche Diagnostics) that quantifies the human β-globin gene. All samples from each woman were tested in the same assay run and results were expressed as the number of DNA copies/10^6 ^CD4^+ ^T cells tested.

### Statistical analysis

The correlations between variables were analyzed by Spearman's rank test. Results were compared using the Mann-Whitney U paired test. *P *values < 0.05 were considered statistically significant. A value equal to half the threshold was allocated to undetectable supernatants (ie 30 HIV-1 RNA copies/ml).

## Competing interests

The authors declare that they have no competing interests.

## Authors' contributions

All authors read and approved the final manuscript.

Designed the experiments/study: DV, ET, YA, FR, PAR, NM, VF, KB, PVP JPV. Enrolledpatients: DV, FR; Collected the data: DV, ET, DV, KB, VF. Analyzed the data: DV, ET, YA, PAR, VF, PVP, JPV. Wrote the first draft of the paper: DV, ET, JPV. Contributed to the writing of the paper: DV, ET, YA, PAR, VF, PVP, JPV.
